# (±)-3-Benz­yloxy-1-(4-meth­oxy­benz­yl)piperidine-2-thione

**DOI:** 10.1107/S1600536812048854

**Published:** 2012-12-05

**Authors:** Daniel P. Pienaar, Sanaz Khorasani, Charles B. de Koning, Joseph P. Michael

**Affiliations:** a Molecular Sciences Institute, School of Chemistry, University of the Witwatersrand, PO Wits 2050, Johannesburg, South Africa

## Abstract

The title mol­ecule, C_20_H_23_NO_2_S, adopts a twisted conformation in which the two aromatic rings connected to the central piperidine ring are orientated *trans* to each other. An intra­molecular C—H⋯S contact occurs. In the crystal, C—H⋯π and C—H⋯O inter­actions act to stabilize the structure in three dimensions.

## Related literature

For the use of related piperidine­thio­nes in the synthesis of febrifugine analogues, see: Michael *et al.* (2006 ▶). For information on the biological activity of febrifugine, see: Murata *et al.* (1998 ▶).
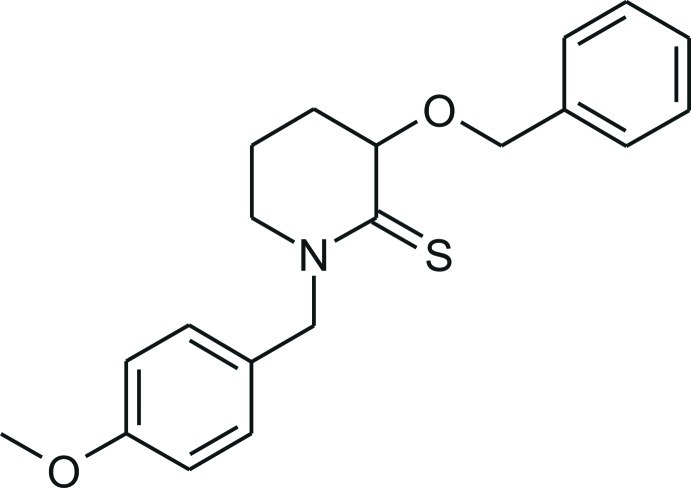


## Experimental

### 

#### Crystal data

C_20_H_23_NO_2_S
*M* *_r_* = 341.45
Orthorhombic, 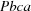

*a* = 18.371 (3) Å
*b* = 10.4844 (15) Å
*c* = 18.467 (3) Å
*V* = 3556.9 (9) Å^3^
*Z* = 8
Mo *K*α radiation
μ = 0.19 mm^−1^
*T* = 173 K
0.47 × 0.28 × 0.05 mm

#### Data collection

Bruker APEXII CCD area-detector diffractometer
22717 measured reflections
4286 independent reflections
2949 reflections with *I* > 2σ(*I*)
*R*_int_ = 0.046

#### Refinement

*R*[*F* ^2^ > 2σ(*F* ^2^)] = 0.037
*wR*(*F* ^2^) = 0.098
*S* = 1.01
4286 reflections
218 parameters
H-atom parameters constrained
Δρ_max_ = 0.26 e Å^−3^
Δρ_min_ = −0.26 e Å^−3^

### 

Data collection: *APEX2* (Bruker, 2005 ▶); cell refinement: *SAINT-NT* (Bruker, 2005 ▶); data reduction: *SAINT-NT*; program(s) used to solve structure: *SHELXS97* (Sheldrick, 2008 ▶); program(s) used to refine structure: *SHELXL97* (Sheldrick, 2008 ▶); molecular graphics: *ORTEP-3 for Windows* (Farrugia, 2012 ▶) and *SCHAKAL99* (Keller, 1999 ▶); software used to prepare material for publication: *WinGX* (Farrugia, 2012 ▶) and *PLATON* (Spek, 2009 ▶).

## Supplementary Material

Crystal structure: contains datablock(s) global, I. DOI: 10.1107/S1600536812048854/go2079sup1.cif

Structure factors: contains datablock(s) I. DOI: 10.1107/S1600536812048854/go2079Isup2.hkl

Supplementary material file. DOI: 10.1107/S1600536812048854/go2079Isup3.cml

Additional supplementary materials:  crystallographic information; 3D view; checkCIF report

## Figures and Tables

**Table 1:** Hydrogen-bond geometry (Å, °) *Cg*1 is the centroid of the C16–C21 ring.

*D*—H⋯*A*	*D*—H	H⋯*A*	*D*⋯*A*	*D*—H⋯*A*
C7—H7*A*⋯S1	0.99	2.54	3.0760 (16)	114
C13—H13⋯O2^i^	0.95	2.59	3.4913 (19)	158
C6—H6*B*⋯*Cg*1^i^	0.99	2.54	3.5066 (19)	165
C14—H14*A*⋯*Cg*1^ii^	0.98	2.61	3.455 (2)	144

Symmetry codes: (i) 
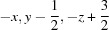
; (ii) 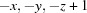.

**Fig. 1.**
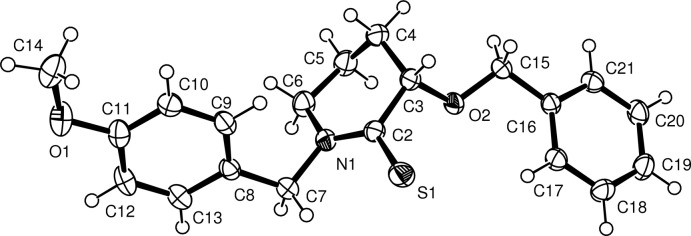
The molecular structure of (I), showing the atomic numbering scheme. Displacement ellipsoids are drawn at the 50% probability level.

**Fig. 2.**
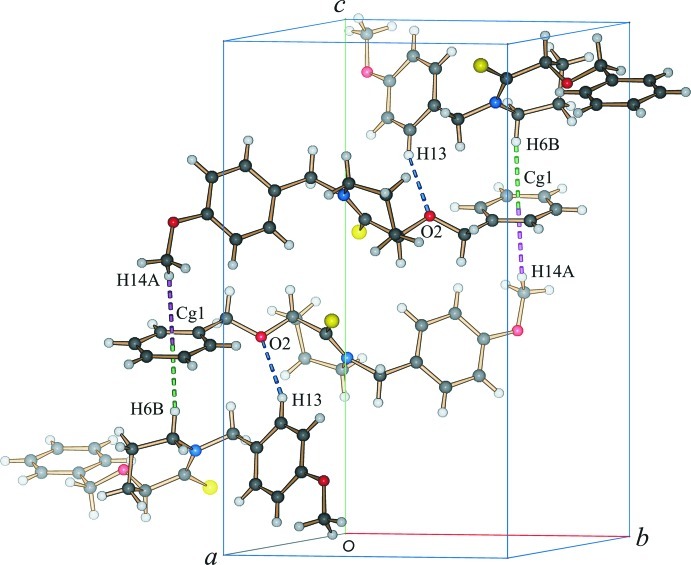
C—H···π and C—H···O interactions in the structure of (I). Only the aromatic ring defined by C16—C21 is involved in C—H···π interactions but these act to bring three molecules together.

## supplementary crystallographic information

### Comment

The title piperidinethione was prepared as an intermediate for the total synthesis of febrifugine, a quinazoline alkaloid with potent antimalarial activity (Murata *et al.*, 1998). Related thiolactam intermediates have been used in the synthesis of febrifugine analogues in ongoing investigations in our laboratories (Michael *et al.*, 2006). It should be noted that, although an optically pure lactam was used in the synthesis of the title compound, racemization took place during the replacement of oxygen by sulfur with Lawesson's reagent.

The title organic compound (Fig. 1) crystallizes in the space group *Pbca*. The molecule adopts a twisted conformation in which the two aromatic rings connected to the piperidine ring are orientated *trans* to each other. The aromatic rings are also rotated with respect to each other such that the angle between least squares planes defined by the two rings is 59.04 (6)°. The most significant weak interactions in this structure are listed in Table 1. Two C—H···π interactions involving the ring defined by C16—C21 are present in the structure while no such interactions exist for the aromatic ring defined by C8—C13. These two C—H···π interactions act to bring three molecules together which interact further through the C—H···O interaction as shown in Fig. 2. No significant π···π interactions are present in the structure.

### Experimental

The title compound was synthesized by heating a mixture of (3*S*)-3-benzyloxy-1-(4-methoxybenzyl)piperidin-2-one (170 mg, 0.52 mmol) and Lawesson's reagent (106 mg, 0.26 mmol) in benzene (8 ml) under reflux for 4 h. After evaporation of the solvent *in vacuo*, the residue was purified by column chromatography on silica gel with hexane/ethyl acetate (4:1 *v*/*v*) as eluent to yield the racemic product as shiny colourless plates (174 mg, 98%), m.p. 349.5–351.5 K.

### Refinement

All H atoms attached to carbon were positioned geometrically, and allowed to ride on their parent atoms, with C—H bond lengths of 0.95 Å (CH), 0.99 Å (CH_2_) or 0.98 Å (CH_3_), and isotropic displacement parameters set to 1.2 (CH and CH_2_) or 1.5 times (CH_3_) the *U*_eq_ of the parent atom.

### Crystal data

**Table:**

C_20_H_23_NO_2_S	*F*(000) = 1456
*M**_r_* = 341.45	*D*_x_ = 1.275 Mg m^−^^3^
Orthorhombic, *P**b**c**a*	Mo *K*α radiation, λ = 0.71073 Å
Hall symbol: -P 2ac 2ab	Cell parameters from 978 reflections
*a* = 18.371 (3) Å	θ = 2.5–28.0°
*b* = 10.4844 (15) Å	µ = 0.19 mm^−^^1^
*c* = 18.467 (3) Å	*T* = 173 K
*V* = 3556.9 (9) Å^3^	Plate, colourless
*Z* = 8	0.47 × 0.28 × 0.05 mm

### Data collection

**Table:**

Bruker APEXII CCD area-detector diffractometer	2949 reflections with *I* > 2σ(*I*)
Radiation source: fine-focus sealed tube	*R*_int_ = 0.046
Graphite monochromator	θ_max_ = 28.0°, θ_min_ = 2.2°
phi and ω scans	*h* = −24→19
22717 measured reflections	*k* = −13→13
4286 independent reflections	*l* = −23→24

### Refinement

**Table:**

Refinement on *F*^2^	Primary atom site location: structure-invariant direct methods
Least-squares matrix: full	Secondary atom site location: difference Fourier map
*R*[*F*^2^ > 2σ(*F*^2^)] = 0.037	Hydrogen site location: inferred from neighbouring sites
*wR*(*F*^2^) = 0.098	H-atom parameters constrained
*S* = 1.01	*w* = 1/[σ^2^(*F*_o_^2^) + (0.0464*P*)^2^ + 0.5277*P*] where *P* = (*F*_o_^2^ + 2*F*_c_^2^)/3
4286 reflections	(Δ/σ)_max_ = 0.003
218 parameters	Δρ_max_ = 0.26 e Å^−^^3^
0 restraints	Δρ_min_ = −0.26 e Å^−^^3^

### Special details

**Table:**

Geometry. All e.s.d.'s (except the e.s.d. in the dihedral angle between two l.s. planes) are estimated using the full covariance matrix. The cell e.s.d.'s are taken into account individually in the estimation of e.s.d.'s in distances, angles and torsion angles; correlations between e.s.d.'s in cell parameters are only used when they are defined by crystal symmetry. An approximate (isotropic) treatment of cell e.s.d.'s is used for estimating e.s.d.'s involving l.s. planes.
Refinement. Refinement of *F*^2^ against ALL reflections. The weighted *R*-factor *wR* and goodness of fit *S* are based on *F*^2^, conventional *R*-factors *R* are based on *F*, with *F* set to zero for negative *F*^2^. The threshold expression of *F*^2^ > σ(*F*^2^) is used only for calculating *R*-factors(gt) *etc*. and is not relevant to the choice of reflections for refinement. *R*-factors based on *F*^2^ are statistically about twice as large as those based on *F*, and *R*- factors based on ALL data will be even larger.

### Fractional atomic coordinates and isotropic or equivalent isotropic displacement parameters (Å^2^)

**Table:**

	*x*	*y*	*z*	*U*_iso_*/*U*_eq_	
C2	−0.00224 (8)	0.06796 (14)	0.61154 (8)	0.0260 (3)	
C3	0.02325 (8)	0.19576 (13)	0.58013 (8)	0.0278 (3)	
H3	0.0100	0.1991	0.5277	0.033*	
C4	0.10388 (8)	0.21947 (16)	0.58763 (9)	0.0339 (4)	
H4A	0.1153	0.3082	0.5733	0.041*	
H4B	0.1311	0.1611	0.5553	0.041*	
C5	0.12655 (9)	0.19740 (15)	0.66565 (9)	0.0371 (4)	
H5A	0.1790	0.2166	0.6715	0.045*	
H5B	0.0988	0.2548	0.6980	0.045*	
C6	0.11198 (8)	0.06000 (15)	0.68586 (9)	0.0339 (4)	
H6A	0.1511	0.0060	0.6654	0.041*	
H6B	0.1140	0.0518	0.7392	0.041*	
C7	0.02107 (8)	−0.11296 (14)	0.69241 (8)	0.0310 (3)	
H7A	−0.0293	−0.1356	0.6782	0.037*	
H7B	0.0225	−0.1057	0.7458	0.037*	
C8	0.07247 (8)	−0.21843 (14)	0.66849 (8)	0.0265 (3)	
C9	0.09864 (8)	−0.22570 (14)	0.59808 (8)	0.0293 (3)	
H9	0.0846	−0.1624	0.5640	0.035*	
C10	0.14501 (8)	−0.32359 (14)	0.57614 (8)	0.0295 (3)	
H10	0.1621	−0.3273	0.5276	0.035*	
C11	0.16592 (8)	−0.41589 (14)	0.62622 (9)	0.0311 (3)	
C12	0.13904 (9)	−0.41094 (14)	0.69669 (9)	0.0353 (4)	
H12	0.1522	−0.4751	0.7306	0.042*	
C13	0.09325 (9)	−0.31299 (14)	0.71751 (9)	0.0324 (4)	
H13	0.0757	−0.3100	0.7659	0.039*	
C14	0.23736 (9)	−0.52714 (17)	0.53823 (10)	0.0434 (4)	
H14A	0.1953	−0.5379	0.5061	0.065*	
H14B	0.2693	−0.6017	0.5342	0.065*	
H14C	0.2643	−0.4503	0.5243	0.065*	
C15	−0.02311 (8)	0.40960 (13)	0.58316 (9)	0.0293 (3)	
H15A	−0.0249	0.3973	0.5300	0.035*	
H15B	0.0205	0.4611	0.5948	0.035*	
C16	−0.09058 (8)	0.47794 (13)	0.60827 (8)	0.0256 (3)	
C17	−0.15243 (8)	0.41094 (14)	0.62983 (8)	0.0300 (3)	
H17	−0.1518	0.3203	0.6303	0.036*	
C18	−0.21487 (9)	0.47569 (15)	0.65056 (9)	0.0359 (4)	
H18	−0.2567	0.4292	0.6654	0.043*	
C19	−0.21667 (9)	0.60777 (16)	0.64975 (9)	0.0373 (4)	
H19	−0.2596	0.6518	0.6640	0.045*	
C20	−0.15566 (9)	0.67528 (15)	0.62815 (9)	0.0351 (4)	
H20	−0.1569	0.7658	0.6270	0.042*	
C21	−0.09291 (8)	0.61116 (14)	0.60820 (8)	0.0303 (3)	
H21	−0.0510	0.6582	0.5943	0.036*	
N1	0.04038 (6)	0.01121 (11)	0.66005 (6)	0.0267 (3)	
S1	−0.08302 (2)	0.01240 (4)	0.58307 (2)	0.03658 (12)	
O1	0.21278 (6)	−0.51478 (10)	0.61136 (7)	0.0413 (3)	
O2	−0.01884 (6)	0.28903 (9)	0.61845 (5)	0.0299 (2)	

### Atomic displacement parameters (Å^2^)

**Table:**

	*U*^11^	*U*^22^	*U*^33^	*U*^12^	*U*^13^	*U*^23^
C2	0.0271 (8)	0.0236 (7)	0.0273 (8)	0.0015 (6)	0.0041 (6)	−0.0019 (6)
C3	0.0297 (8)	0.0245 (7)	0.0293 (8)	0.0041 (6)	0.0051 (6)	0.0001 (6)
C4	0.0289 (8)	0.0281 (8)	0.0447 (10)	0.0004 (6)	0.0072 (7)	0.0032 (7)
C5	0.0297 (9)	0.0303 (9)	0.0514 (10)	−0.0019 (7)	−0.0028 (7)	−0.0028 (7)
C6	0.0296 (8)	0.0319 (8)	0.0402 (9)	−0.0004 (7)	−0.0079 (7)	−0.0001 (7)
C7	0.0320 (8)	0.0288 (8)	0.0321 (8)	0.0011 (6)	0.0032 (7)	0.0063 (6)
C8	0.0274 (8)	0.0219 (7)	0.0303 (8)	−0.0025 (6)	−0.0027 (6)	0.0021 (6)
C9	0.0318 (8)	0.0239 (7)	0.0322 (8)	−0.0015 (6)	−0.0036 (6)	0.0049 (6)
C10	0.0311 (8)	0.0270 (8)	0.0303 (8)	−0.0038 (6)	−0.0010 (6)	−0.0019 (6)
C11	0.0304 (8)	0.0228 (8)	0.0402 (9)	0.0001 (6)	−0.0102 (7)	−0.0045 (6)
C12	0.0468 (10)	0.0243 (8)	0.0348 (9)	0.0027 (7)	−0.0128 (7)	0.0044 (7)
C13	0.0413 (9)	0.0277 (8)	0.0282 (8)	−0.0028 (7)	−0.0036 (7)	0.0030 (6)
C14	0.0348 (9)	0.0402 (10)	0.0552 (11)	0.0066 (7)	−0.0036 (8)	−0.0135 (8)
C15	0.0287 (8)	0.0220 (7)	0.0371 (9)	0.0003 (6)	0.0033 (6)	0.0049 (6)
C16	0.0258 (7)	0.0222 (7)	0.0290 (7)	−0.0001 (6)	−0.0030 (6)	0.0012 (6)
C17	0.0298 (8)	0.0212 (7)	0.0390 (9)	−0.0024 (6)	−0.0011 (6)	0.0005 (6)
C18	0.0261 (8)	0.0327 (9)	0.0488 (10)	−0.0036 (7)	0.0016 (7)	0.0014 (7)
C19	0.0308 (9)	0.0337 (9)	0.0473 (10)	0.0083 (7)	0.0001 (7)	−0.0015 (7)
C20	0.0404 (10)	0.0209 (8)	0.0440 (10)	0.0037 (7)	−0.0011 (7)	−0.0001 (7)
C21	0.0314 (8)	0.0239 (8)	0.0356 (8)	−0.0043 (6)	0.0001 (6)	0.0024 (6)
N1	0.0265 (6)	0.0233 (6)	0.0303 (7)	0.0009 (5)	0.0005 (5)	0.0009 (5)
S1	0.0307 (2)	0.0367 (2)	0.0423 (2)	−0.00675 (17)	−0.00588 (17)	0.00665 (18)
O1	0.0433 (7)	0.0328 (6)	0.0479 (7)	0.0116 (5)	−0.0118 (5)	−0.0078 (5)
O2	0.0343 (6)	0.0235 (5)	0.0318 (6)	0.0065 (4)	0.0075 (5)	0.0037 (4)

### Geometric parameters (Å, º)

**Table:**

C2—N1	1.3302 (19)	C11—O1	1.3752 (19)
C2—C3	1.533 (2)	C11—C12	1.393 (2)
C2—S1	1.6788 (15)	C12—C13	1.382 (2)
C3—O2	1.4334 (17)	C12—H12	0.9500
C3—C4	1.509 (2)	C13—H13	0.9500
C3—H3	1.0000	C14—O1	1.430 (2)
C4—C5	1.518 (2)	C14—H14A	0.9800
C4—H4A	0.9900	C14—H14B	0.9800
C4—H4B	0.9900	C14—H14C	0.9800
C5—C6	1.512 (2)	C15—O2	1.4244 (17)
C5—H5A	0.9900	C15—C16	1.505 (2)
C5—H5B	0.9900	C15—H15A	0.9900
C6—N1	1.4897 (19)	C15—H15B	0.9900
C6—H6A	0.9900	C16—C17	1.394 (2)
C6—H6B	0.9900	C16—C21	1.397 (2)
C7—N1	1.4756 (18)	C17—C18	1.387 (2)
C7—C8	1.520 (2)	C17—H17	0.9500
C7—H7A	0.9900	C18—C19	1.385 (2)
C7—H7B	0.9900	C18—H18	0.9500
C8—C9	1.388 (2)	C19—C20	1.384 (2)
C8—C13	1.396 (2)	C19—H19	0.9500
C9—C10	1.394 (2)	C20—C21	1.384 (2)
C9—H9	0.9500	C20—H20	0.9500
C10—C11	1.393 (2)	C21—H21	0.9500
C10—H10	0.9500		
			
N1—C2—C3	117.77 (13)	O1—C11—C10	124.33 (15)
N1—C2—S1	125.17 (12)	C12—C11—C10	119.78 (14)
C3—C2—S1	117.04 (11)	C13—C12—C11	120.22 (14)
O2—C3—C4	111.83 (12)	C13—C12—H12	119.9
O2—C3—C2	104.16 (11)	C11—C12—H12	119.9
C4—C3—C2	114.15 (12)	C12—C13—C8	120.93 (15)
O2—C3—H3	108.8	C12—C13—H13	119.5
C4—C3—H3	108.8	C8—C13—H13	119.5
C2—C3—H3	108.8	O1—C14—H14A	109.5
C3—C4—C5	109.36 (13)	O1—C14—H14B	109.5
C3—C4—H4A	109.8	H14A—C14—H14B	109.5
C5—C4—H4A	109.8	O1—C14—H14C	109.5
C3—C4—H4B	109.8	H14A—C14—H14C	109.5
C5—C4—H4B	109.8	H14B—C14—H14C	109.5
H4A—C4—H4B	108.3	O2—C15—C16	109.08 (12)
C6—C5—C4	109.33 (13)	O2—C15—H15A	109.9
C6—C5—H5A	109.8	C16—C15—H15A	109.9
C4—C5—H5A	109.8	O2—C15—H15B	109.9
C6—C5—H5B	109.8	C16—C15—H15B	109.9
C4—C5—H5B	109.8	H15A—C15—H15B	108.3
H5A—C5—H5B	108.3	C17—C16—C21	118.62 (14)
N1—C6—C5	113.86 (13)	C17—C16—C15	121.30 (13)
N1—C6—H6A	108.8	C21—C16—C15	120.05 (13)
C5—C6—H6A	108.8	C18—C17—C16	120.43 (14)
N1—C6—H6B	108.8	C18—C17—H17	119.8
C5—C6—H6B	108.8	C16—C17—H17	119.8
H6A—C6—H6B	107.7	C19—C18—C17	120.40 (15)
N1—C7—C8	112.02 (12)	C19—C18—H18	119.8
N1—C7—H7A	109.2	C17—C18—H18	119.8
C8—C7—H7A	109.2	C20—C19—C18	119.67 (15)
N1—C7—H7B	109.2	C20—C19—H19	120.2
C8—C7—H7B	109.2	C18—C19—H19	120.2
H7A—C7—H7B	107.9	C19—C20—C21	120.17 (15)
C9—C8—C13	118.27 (14)	C19—C20—H20	119.9
C9—C8—C7	121.82 (13)	C21—C20—H20	119.9
C13—C8—C7	119.89 (13)	C20—C21—C16	120.71 (14)
C8—C9—C10	121.62 (14)	C20—C21—H21	119.6
C8—C9—H9	119.2	C16—C21—H21	119.6
C10—C9—H9	119.2	C2—N1—C7	121.73 (13)
C11—C10—C9	119.16 (14)	C2—N1—C6	125.56 (13)
C11—C10—H10	120.4	C7—N1—C6	112.68 (12)
C9—C10—H10	120.4	C11—O1—C14	117.04 (13)
O1—C11—C12	115.88 (14)	C15—O2—C3	114.16 (11)
			
N1—C2—C3—O2	−101.85 (14)	C21—C16—C17—C18	0.2 (2)
S1—C2—C3—O2	76.83 (14)	C15—C16—C17—C18	−177.91 (15)
N1—C2—C3—C4	20.40 (19)	C16—C17—C18—C19	0.3 (2)
S1—C2—C3—C4	−160.92 (11)	C17—C18—C19—C20	−0.1 (3)
O2—C3—C4—C5	67.16 (16)	C18—C19—C20—C21	−0.7 (3)
C2—C3—C4—C5	−50.78 (17)	C19—C20—C21—C16	1.2 (2)
C3—C4—C5—C6	62.00 (17)	C17—C16—C21—C20	−0.9 (2)
C4—C5—C6—N1	−43.34 (18)	C15—C16—C21—C20	177.18 (14)
N1—C7—C8—C9	−39.1 (2)	C3—C2—N1—C7	−178.97 (12)
N1—C7—C8—C13	142.40 (14)	S1—C2—N1—C7	2.5 (2)
C13—C8—C9—C10	−0.6 (2)	C3—C2—N1—C6	−1.2 (2)
C7—C8—C9—C10	−179.13 (14)	S1—C2—N1—C6	−179.75 (12)
C8—C9—C10—C11	−0.4 (2)	C8—C7—N1—C2	112.23 (15)
C9—C10—C11—O1	−178.26 (14)	C8—C7—N1—C6	−65.82 (16)
C9—C10—C11—C12	1.5 (2)	C5—C6—N1—C2	13.5 (2)
O1—C11—C12—C13	178.11 (14)	C5—C6—N1—C7	−168.55 (13)
C10—C11—C12—C13	−1.7 (2)	C12—C11—O1—C14	175.93 (13)
C11—C12—C13—C8	0.7 (2)	C10—C11—O1—C14	−4.3 (2)
C9—C8—C13—C12	0.4 (2)	C16—C15—O2—C3	155.93 (12)
C7—C8—C13—C12	179.00 (14)	C4—C3—O2—C15	76.17 (15)
O2—C15—C16—C17	−29.49 (19)	C2—C3—O2—C15	−160.07 (12)
O2—C15—C16—C21	152.48 (13)		

### Hydrogen-bond geometry (Å, º)

Cg1 is the centroid of the C16–C21 ring.

**Table:**

*D*—H···*A*	*D*—H	H···*A*	*D*···*A*	*D*—H···*A*
C7—H7*A*···S1	0.99	2.54	3.0760 (16)	114
C13—H13···O2^i^	0.95	2.59	3.4913 (19)	158
C6—H6*B*···*Cg*1^i^	0.99	2.54	3.5066 (19)	165
C14—H14*A*···*Cg*1^ii^	0.98	2.61	3.455 (2)	144

Symmetry codes: (i) −*x*, *y*−1/2, −*z*+3/2; (ii) −*x*, −*y*, −*z*+1.
